# Biodiversity Patterns along Ecological Gradients: Unifying β-Diversity Indices

**DOI:** 10.1371/journal.pone.0110485

**Published:** 2014-10-17

**Authors:** Robert C. Szava-Kovats, Meelis Pärtel

**Affiliations:** Institute of Ecology and Earth Sciences, University of Tartu, Tartu, Estonia; Argonne National Laboratory, United States of America

## Abstract

Ecologists have developed an abundance of conceptions and mathematical expressions to define β-diversity, the link between local (α) and regional-scale (γ) richness, in order to characterize patterns of biodiversity along ecological (i.e., spatial and environmental) gradients. These patterns are often realized by regression of β-diversity indices against one or more ecological gradients. This practice, however, is subject to two shortcomings that can undermine the validity of the biodiversity patterns. First, many β-diversity indices are constrained to range between fixed lower and upper limits. As such, regression analysis of β-diversity indices against ecological gradients can result in regression curves that extend beyond these mathematical constraints, thus creating an interpretational dilemma. Second, despite being a function of the same measured α- and γ-diversity, the resultant biodiversity pattern depends on the choice of β-diversity index. We propose a simple logistic transformation that rids beta-diversity indices of their mathematical constraints, thus eliminating the possibility of an uninterpretable regression curve. Moreover, this transformation results in identical biodiversity patterns for three commonly used classical beta-diversity indices. As a result, this transformation eliminates the difficulties of both shortcomings, while allowing the researcher to use whichever beta-diversity index deemed most appropriate. We believe this method can help unify the study of biodiversity patterns along ecological gradients.

## Introduction

Ecologists have long expressed interest in spatial and environmental effects on biodiversity, specifically the effects with respect to β-diversity, which quantifies the similarity of species assemblage among sites and represents the link between local (α) diversity and regional (γ) diversity [1]. Consensus on a standard definition of β-diversity, however, has been elusive and has been the subject of considerable debate – both in terms of its fundamental essence [1]–[5] and its mathematical relationship with α- and γ-diversity [2], [4], [6]–[8]. Much mathematical discussion revolves on whether β-diversity is defined better by a multiplicative (i.e., 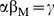) [9] or an additive (i.e., 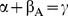) [10], [11] partition.

Over time, many different expressions for “β-diversity” have been proposed, which, in addition to considerable ambiguous terminology, has led to confusion within the ecological community [2], [4], [12]. Although each expression addresses the compositional similarity among sites, each is also a different measure of compositional similarity and thus, describes a somewhat different concept. The choice of beta-diversity index is, therefore, dependent on the researcher's ecological application [4]. One set of beta-diversity indices, the “classical metrics” [1], defines α-diversity in terms of the mean diversity in local sites and γ-diversity as the composite of these local sites.

One popular approach to quantify spatial or environmental patterns of biodiversity is regression analysis of a classical β-diversity index against one or more spatial or environmental gradients [13]–[19]. This approach, however, is subject to two shortcomings that can undermine the validity of the resultant diversity patterns. First, classical β-diversity indices are mathematically constrained, i.e., each index features a lower limit when all local sites are compositionally the same and an upper limit when all local sites are unique. Regression of such β-diversity indices can result in a predicted estimation that crosses these limits, thus violating their mathematical constraints. Such instances cast doubt on the validity of the regression diagnostics, e.g., correlation, residuals, t-tests etc. Second, the regression analysis is dependent on the choice of β-diversity index, thus a biodiversity pattern resultant from one β-diversity index may be radically different from another β-diversity index.

We introduce a simple transformation that rids commonly used classical β-diversity indices of their lower and upper constraints, thereby eliminating the risk of regression analysis producing non-interpretable regression curves. Moreover, regression of these transformed indices against spatial or environmental gradients yields identical relationships for these commonly used classical β-diversity indices. As a result, this transformation eliminates the difficulties of both shortcomings, while allowing the researcher to use whichever β-diversity index deemed most appropriate.

## Methods: Mathematical Properties of Logistic-Transformed β-Diversity Indices

Classical β-diversity indices are derived from the measured local diversity (α) – usually expressed as the arithmetic mean of species richness in *N* local sites – and regional diversity (γ), the measured species richness of all *N* local sites combined. β-diversity is often expressed by one of three β-diversity indices, which are described here using the terminology in Tuomisto [4]. True β-diversity, β_Md_, expressed as 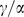, quantifies the number of compositional units in the collection. β_Md_ is equivalent to β_M_, the basic multiplicative diversity partition. β_Md-1_, expressed as 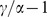, provides the number of complete turnovers among the compositional units in the collection. Proportional species turnover, β_Pt_, expressed as 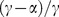, described the proportion of species found regionally that are not found locally.

All three β-diversity indices, β_Md_, β_Md-1_, and β_Pt_, assume a minimum value when all local sites are compositionally identical and a maximum when all local sites are compositionally unique. The lower limit for β_Md-1_ and β_Pt_ is zero and for β_Md_ unity. The respective upper limits are a function of *N* (Table 1).

**Table 1:** Mathematical definitions and expressions of beta-diversity indices.

β-index	Function	Low	High
β_Md_	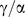	1	*N*
β_Md-1_	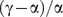	0	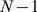
β_Pt_	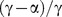	0	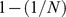

Low and High represent the lower and upper limits of beta-diversity indices as a function of the number of local sites (*N*).

A logistic transformation 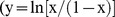 is a standard method by which to treat data that are constrained by upper and lower limits [20]. An analogous transformation applied to these β-diversity indices takes the generalized form, 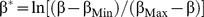, where β_Max_ and β_Min_ are the respective upper and lower limit of a β-diversity index, β. Index β can be recovered from β^*^ by 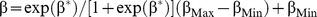. This retransformation allows the depiction of beta-diversity in its more familiar constrained units, as a logistic variable may be difficult to visualize. The beta-diversity relationship with a gradient can be plotted simply as the retransformed β^*^ as a function of the gradient. Any value of β^*^ will — upon retransformation into β — adhere to the mathematical constraint, 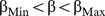. Given, for example, a set of 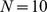 local sites with 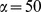 and 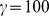, β_Md_, β_Md-1_, and β_Pt_ are 2, 1, and 0.5, respectively. Corresponding values of β^*^_Md_, β^*^_Md-1_, and β^*^_Pt_ are −2.08, −2.08, and 0.22, respectively. Note that because 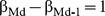, , and that 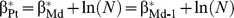.

## Results: Illustrative Example

Table 2 provides a set of illustrative data of α- and γ-diversity along a hypothetical environmental or spatial gradient to compare the performance of these commonly used classical β-diversity indices with their logistic-transformed equivalents. Ten points along the gradient were assigned values of γ and different values of α for three scenarios. In all scenarios, 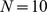, thus the upper limits for β_Md_, β_Md-1_, and β_Pt_ are 10, 9, and 0.9, respectively.

**Table 2:** Values of γ- and α-diversity along a hypothetical ecological gradient for three scenarios.

Gradient		Scenario		
	γ	α(A)	α(B)	α(C)
1	10	9.5	2	9.5
2	20	18.6	6	10
3	40	34	6	38
4	30	18	10.5	9
5	50	37.5	17.5	31.2
6	80	24	12	46.5
7	70	21	8.4	14
8	100	12	12	40
9	80	9.6	12	24
10	90	13.5	9.9	35.8

Number of local sites (*N*)  = 10.

**Figure 1:**
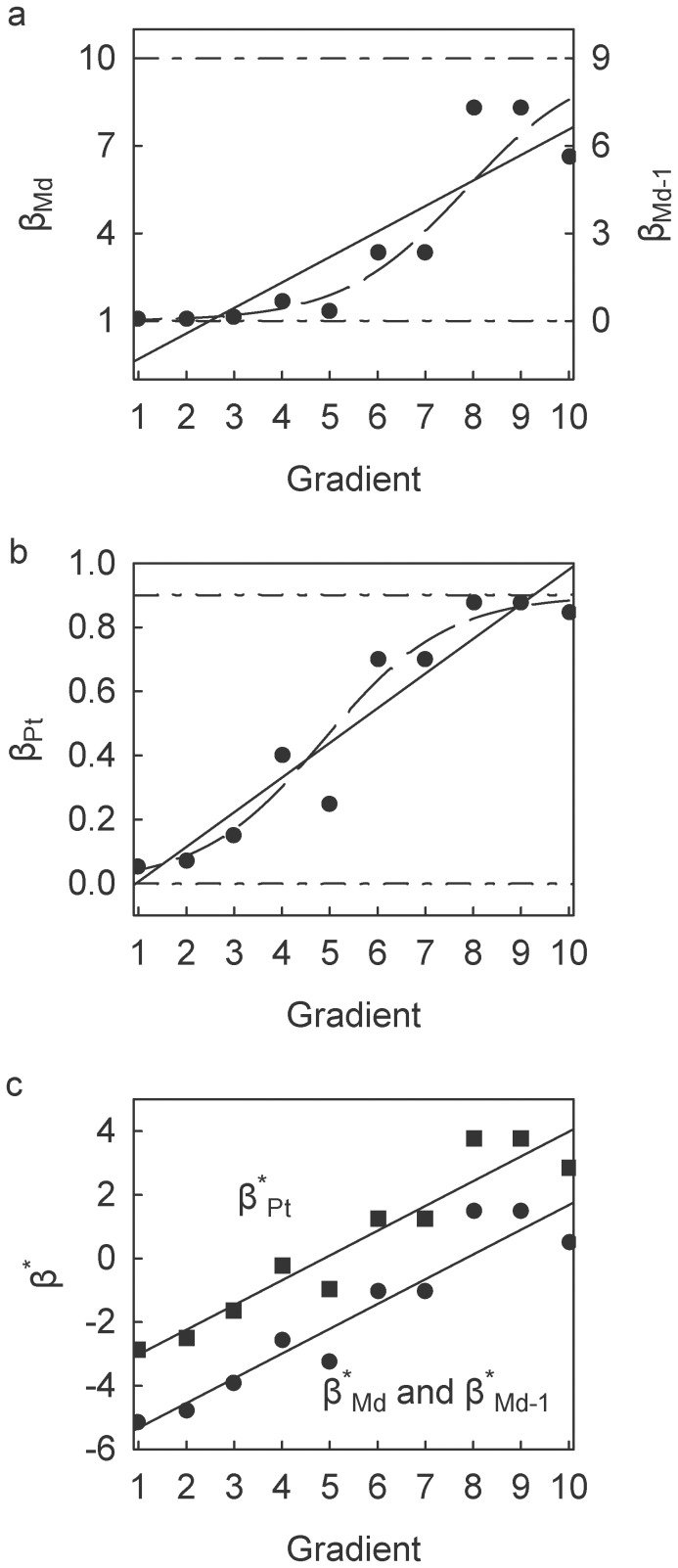
Scatterplots of beta-diversity indices against hypothetical ecological gradient for Scenario A. (a) β_Md_ (left axis) and β_Md-1_ (right axis); linear regression trends, β_Md_ 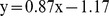, for β_Md-1_ 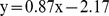 (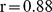 for both). (b) β_Pt_; linear regression trend, 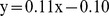 (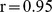). (c) β^*^_Md_, β^*^_Md-1_ (circles) and β^*^_Pt_ (squares); linear regression trends, for β^*^_Md_ β^*^_Md-1_ 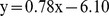, for β^*^_Pt_ 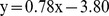 (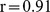 for all). Dashed trends in (a) and (b) depict linear trends of β^*^_Md_ (and β^*^_Md-1_) and β^*^_Pt_ retransformed to β_Md_ (and β_Md-1_) and β_Pt_, respectively. See Table 1 for description of beta-diversity indices and Table 2 for data for Scenario A.

In Scenario A, β_Md_, β_Md-1_, and β_Pt_ all exhibit a statistically significant positive relationship along the gradient. The linear regression curves of β_Md_ and β_Md-1_ cross below the lower limits, 1 and 0 respectively, at gradient values 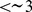 (Figure 1a). The linear regression curve of β_Pt_, derived from the same set of α and γ, crosses the upper limit at gradient values 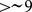 (Figure 1b). In other words, Scenario A provides an example of two different β-diversity indices violating their mathematical constraints at opposite ends of the gradient. The logistic-transformed equivalents, β^*^_Md_, β^*^_Md-1_, and β^*^_Pt_, when regressed along the gradient yield statistically significant linear relationships with identical values for all regression parameters except for the intercept (Figure 1c). The intercept of β^*^_Pt_ is 2.30 greater than that of β^*^_Md_ and β^*^_Md-1_, which is equivalent to exp(*N*), i.e., exp(10). Moreover, the resultant regression curves when retransformed into their original β-diversity indices adhere to their respective upper and lower constraints (Figure 1a,b).

**Figure 2:**
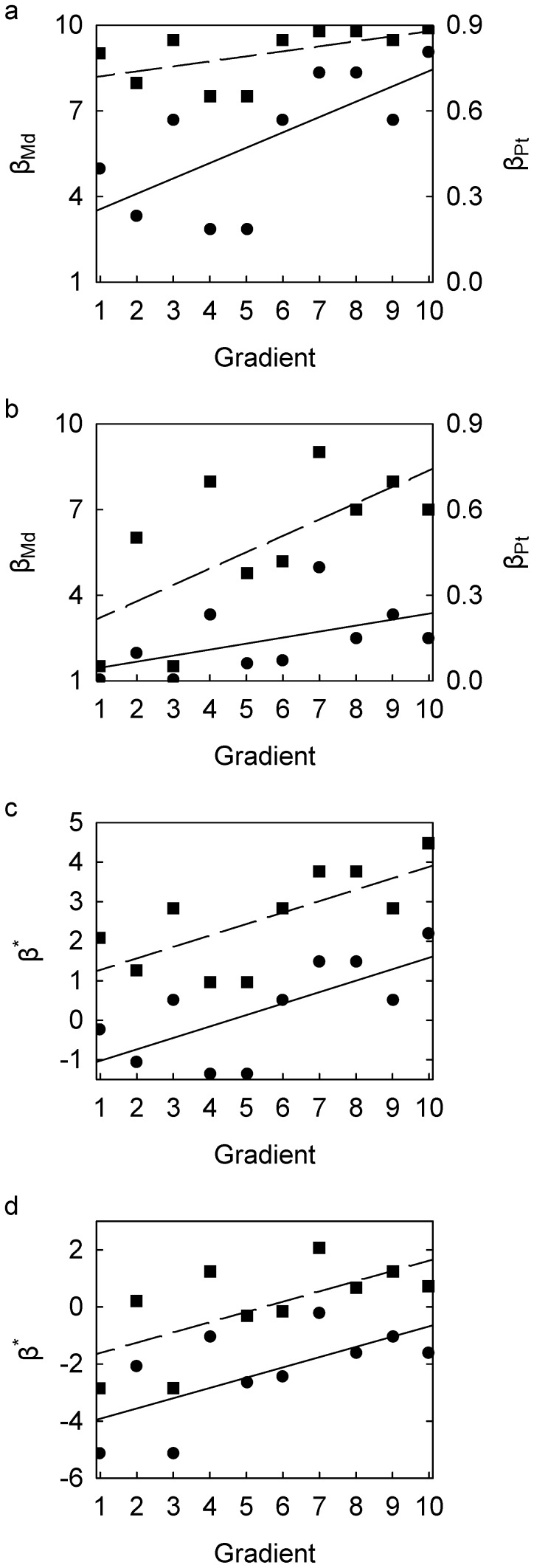
Scatterplots of beta-diversity indices against hypothetical ecological gradient. (a) Scenario B: β_Md_ (circles, left axis) and β_Pt_ (squares, right axis); linear regression trends, for β_Md_ 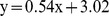 (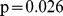), for β_Pt_ 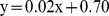 (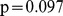). (b) Scenario C: β_Md_ (circles, left axis) and β_Pt_ (squares, right axis); linear regression trends, for β_Md_ 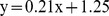 (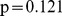), for β_Pt_ 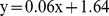 (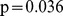). (c) Scenario B: β^*^_Md_ (circles) and β^*^_Pt_ (squares); linear regression trends, for β^*^_Md_ 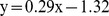, for β^*^_Pt_ 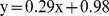 (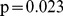 for both). (d) Scenario C: β^*^_Md_ (circles) and β^*^_Pt_ (squares); linear regression trends, for β^*^_Md_ 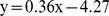, for β^*^_Pt_ 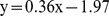 (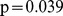 for both). See Table 1 for description of beta-diversity indices and Table 2 for data for Scenario A.

The linear regression curves of β_Md_, β_Md-1_, and β_Pt_ in Scenarios B and C all lie within the upper and lower limits. However, the relationship between β_Pt_ and the gradient in Scenario B is statistically significant at 95% confidence (p = 0.026), whereas those between β_Md_ and β_Md-1_ are non-significant (p = 0.097) (Figure 2a). This result is opposite in Scenario C: the relationship between β_Pt_ and the gradient is statistically non-significant (p = 0.121), whereas those between β_Md_ and β_Md-1_ are significant (p = 0.036) (Figure 2b). In addition, the magnitude of the residuals in Scenarios B and C differ between β_Md_ (or β_Md-1_) and β_Pt_. By contrast, linear regression of β^*^_Md_, β^*^_Md-1_, and β^*^_Pt_ against the gradient results in identical models for both Scenario B and C with the exception of their respective intercepts. The relationship is significant for both Scenario B (p = 0.023) and Scenario C (p = 0.039) and both relationships are independent of the choice of beta-diversity index. In addition, this choice has no effect on the resultant residual patterns (Figure 2c,d).

## Discussion

Space structure is an often neglected consideration in statistical analysis. All conventional multivariate statistical analysis assume that the data occupy Euclidean space [21], yet this is rarely the case. A constrained space structure can usually be readily transformed into Euclidean space; log-transformation of positive-only data is a well-known example. Likewise, logistic transformation can be used to place data constrained by upper and lower limits into an unbounded line in Real space. Logistic transformation is commonly used for data restricted to values between one and unity (as in odds ratios) [20], [22], but can also be performed on constrained classical β-diversity indices. As our illustrative example shows, regression analysis of “raw” classical β-diversity indices can result in regression curves that violate their imposed mathematical constraints, thereby undermining the validity of the regression model and its interpretation. By contrast, regression of logistic-transformed β-diversity indices excludes the possibility of violating these mathematical constraints, thereby eliminating any “impossible” results that would undermine the regression model.

Logistic transformation also eliminates the effects of the choice of these classical β-diversity indices. Although regression of β_Md_ and β_Md-1_ results in identical diversity patterns (the intercept using β_Md-1_ is one less than that using β_Md_), neither is equivalent to β_Pt_, which results in a different diversity pattern. This dilemma is especially worrisome; it seems intuitive that a single measured set of α- and γ-diversity should lead to a unique diversity pattern even if β-diversity is expressed by different indices. Our illustrative example shows that a diversity pattern along an ecological gradient can be statistically significant using one index and non-significant using another. Even if the interpretational contrast is not so dramatic, the difference among β-diversity indices can still affect the residuals, which can influence outlier detection, particularly when data points lie close to the limits. By contrast, logistic transformation results in a unique diversity pattern for all three logistic-transformed indices. The difference between β^*^_Md_ (and β^*^_Md-1_) and β^*^_Pt_ is simply a function of the number of local sites. As a result, all regression parameters (except the intercept) are identical for all β-diversity indices.

Although the illustrative examples shown reflect simple linear regression relationships, the two advantages of the logistic transformation are maintained regardless of the actual relationship, i.e. even if the relationship between logistic-transformed beta-diversity and ecological gradient is markedly non-linear. As a result, the researcher is free to choose any appropriate regression model, including many non-linear models, to describe the beta-diversity pattern. For instance, a polynomial or piecewise (i.e., segmented) regression model could be used to characterize a unimodal relationship. Positive-only regression models, such as logarithmic and exponential models, are excluded, because logistic beta-diversity indices can assume negative values. The lack of equivalency among the “raw” classical beta-diversity indices can lead to inconsistency in describing even the qualitative nature of biodiversity patterns. For example, whereas the relationship of β_Pt_ with the gradient in Figure 2b is linear, the relationship with β_Md_ and β_Md-1_ is arguably non-linear (Figure 2a). As a result, using β_Md_ or β_Md-1_ could lead to the conclusion that beta diversity increases only along the upper part of the gradient, whereas using β_Pt_ suggests that beta-diversity increases along the entire gradient.

The lack of equivalency notwithstanding, attempts to circumvent the constraints on classical β-diversity indices by other methods are fraught with difficulty. For instance, the arc-sine transformation, despite its long tradition of use to mitigate constrained data such as percentage data, has been the subject of criticism and is rapidly running out of favour [22]–[24]. Moreover, the results of regression on arc-sine transformed indices remains dependent on the choice of index. Regression of logistic-transformed indices typically presumes that the data contain no regions in which all the local sites are either unique or identical; logistic transformation is impossible for a data point located exactly on an upper or lower limit. However, methods are available to perform regression analysis based on a logistic-transformed response variable that includes otherwise non-transformable data [25], [26].

Although we have demonstrated the use of logistic transformation on the regression of classical β-diversity indices, the transformation can also be used to circumvent violation of the constraints of pairwise “multivariate measures” [1], such as the Jaccard and Sørensen indices, which are constrained to values between 0 and 1. The Jaccard and Sørensen indices are equivalent to β_Pt_ and β_Md-1_, respectively, for a pair of sites. As such, the logistic transformation can also result in a unique diversity pattern for the Jaccard and Sørensen indices. Logistic-transformation of β-diversity indices is not limited to simple presence-absence estimates with α-diversity calculated as the arithmetic mean. A similar approach can be followed by expressing α-diversity as a geometric mean, as the maximum richness in a set of local sites [27], [28], or by expressing diversity in terms of “effective numbers of species”, which incorporates species abundance [29]–[31]. “Effective numbers of species” can be calculated from, for instance, Shannon or Simpson indices. Moreover, the method can also be applied to phylogenetic and functional richness [32]. We suggest the logical transformation of β-diversity indices as a means of improving and simplifying the interpretation of diversity patterns along ecological gradients.

## References

[1] Anderson MJ, Crist TO, Chase JM, Vellend M, Inouye BD, et al (2011) Navigating the multiple meanings of β diversity: a roadmap for the practicing ecologist. Ecology Letters 14: 19–28. 21070562 10.1111/j.1461-0248.2010.01552.x

[2] Jurasinski G, Koch M (2011) Commentary: do we have a consistent terminology for species diversity? We are on the way. Oecologia 167: 893–902. 21938639 10.1007/s00442-011-2126-6

[3] Jurasinski G, Retzer V, Beierkuhnlein C (2009) Inventory, differentiation, and proportional diversity: a consistent terminology for quantifying species diversity. Oecologia 159: 15–26. 18953572 10.1007/s00442-008-1190-z

[4] Tuomisto H (2010) A diversity of beta diversities: straightening up a concept gone awry. Part 1. Definging beta diversity as a function of alpha and gamma diversity. Ecography 33: 2–22.

[5] Legendre P, De Cáceras M (2013) Beta diversity as the variance of community data: dissimilarity coefficients and partitioning. Ecology Letters 16: 951–963. 23809147 10.1111/ele.12141

[6] Ellison AM (2010) Partitioning diversity. Ecology 91: 1962–1963. 20715615 10.1890/09-1692.1

[7] Ricotta C (2010) On beta diversity decomposition: Trouble shared is not trouble halved. Ecology 91: 1981–1983. 20715619 10.1890/09-0126.1

[8] Veech JA, Summerville KS, Crist TO, Gering JC (2002) The additive partitioning of species diversity: recent revival of an old idea. Oikos 99: 3–9.

[9] Whittaker RH (1960) Vegetation of the Siskiyou Mountains, Oregon and California. Ecological Monographs 30: 279–338.

[10] Lande R (1996) Statistics and partitioning of species diversity, and similarity among multiple communities. Oikos 76: 5–13.

[11] MacArthur R, Recher H, Cody M (1966) On the relation between habitat selection and species diversity. American Naturalist 100: 319–332.

[12] Tuomisto H (2010) A consistent terminology for quantifying species diversity? Yes, it does exist. Oecologia 164: 853–860. 20978798 10.1007/s00442-010-1812-0

[13] Angeler DG, Drakare S (2013) Tracing alpha, beta, and gamma diversity responces to environmental change in boreal lakes. Oecologia 172: 1191–1202. 23229393 10.1007/s00442-012-2554-y

[14] Janišová M, Michalcová D, Bacaro G, Ghisla A (2014) Landscape effects on diversity of semi-natural grasslands. Agriculture, Ecosystems and Environment 182: 47–58.

[15] Lezama F, Santiago B, Altesor A, Cesa A, Chaneton EJ, et al (2014) Variation of grazing-induced vegetation changes across a large-scale productivity gradient. Journal of Vegetation Science 25: 8–21.

[16] Mori AS, Shiono T, Koide D, Kitagawa R, Ota AT, et al (2013) Community assembly processes shape an altitudinal gradient of forest biodiversity. Global Ecology and Biogeography 22: 878–888.

[17] Qian H, Chen S, Mao L, Ouyang Z (2013) Divers of β-diversity along latitudinal gradients revisited. Global Ecology and Biogeography 22: 659–670.

[18] Qian H, Jong-Suk S (2013) Latitudinal gradients of associations between beta and gamma diversity of trees in forest communities in the New World. Journal of Plant Ecology 6: 12–18.

[19] Rocha-Ortega M, Favila ME (2013) The recovery of ground ant diversity in secondary Lacandon tropical forests. Journal of Insect Conservation 17: 1161–1167.

[20] Bolker BM (2008) Ecological Models and Data in R. Princeton, NJ: Princeton University Press. 396 p.

[21] Bacon-Shone J (2011) A short history of compositional data analysis: Theory and Applications. In: V Pawlowsky-Glahn and A Buccianti, editors.Compositional Data Analysis.West Sussex: John Wiley & Sons, Ltd. pp.3–11.

[22] Wilson K, Hardy CW (2002) Statistical analysis of sex ratios: an introduction. In: I. C. W Hardy, editor editors.Sex Ratios: Concepts and Research Methods.Cambridge: Cambridge University Press. pp.48–92.

[23] Milligan GW (1987) The use of the Arc-Sine Transformation in the Analysis of Variance. Educational and Psychological Management 47: 563–573.

[24] Warton DI, Hui FKC (2011) The arcsine is asinine: the analysis of proportions in ecology. Ecology 92: 3–10. 21560670 10.1890/10-0340.1

[25] Papke LE, Wooldridge JM (1996) Econometric methods for fractional response variables with an application to 401(k) plan participation rates. Journal of Applied Economoetrics 11: 619–632.

[26] Ramalho EA, Ramalho JJS, Murteira JMR (2011) Alternative estimating and testing empirical strategies for fractional regression models. Journal of Economic Surveys 25: 19–68.

[27] Harrison S, Ross SJ, Lawton JH (1992) Beta diversity on geographic gradients in Britain. Journal of Animal Ecology 61: 151–158.

[28] Magurran AE (2004) Measuring Biological Diversity.. Oxford: Blackwell Publishing. 256 p.

[29] Hill MO (1973) Diversity and evenness: a unifying notation and its consequences. Ecology 54: 427–432.

[30] Jost L (2006) Entropy and diversity. Oikos 113: 363–375.

[31] Jost L (2007) Partitioning diversity into independent alpha and beta components. Ecology 88: 2427–2439. 18027744 10.1890/06-1736.1

[32] Scheiner SM (2012) A metric of biodiversity that integrates abundance, phylogeny, and function. Oikos 121: 1191–1202.

